# Diagnostic Challenge of Acute Hepatitis in Human Immunodeficiency Virus-Positive Patients: With or Without Tuberculosis

**DOI:** 10.7759/cureus.16449

**Published:** 2021-07-17

**Authors:** Cristel M Rodriguez-Vargas, Nicole Vergara, Ana B Arauz

**Affiliations:** 1 Internal Medicine, Hospital Santo Tomás, Panama, PAN; 2 Pathology, Hospital Santo Tomás, Panama, PAN; 3 Infectious Diseases, Hospital Santo Tomás, Panama, PAN; 4 Internal Medicine, Universidad de Panamá, Panama, PAN

**Keywords:** tuberculosis, hepatic tuberculosis, hiv infection, multidrug-resistant tuberculosis, miliary tuberculosis

## Abstract

Tuberculosis (TB) is the leading infectious disease that causes death worldwide, eclipsing HIV/AIDS. It may affect any organ, but the most common manifestation is related to the involvement of the lungs. Hepatic tuberculosis is often a manifestation of disseminated disease and less likely a localized disease. Our case illustrates an HIV-positive patient with disseminated tuberculosis that presented first as liver involvement. After the diagnosis was made through liver biopsy, pulmonary compromise ensued. We review the clinical presentation, diagnosis, and treatment options of disseminated and hepatic TB. Our case is a glimpse of the many faces TB can adopt, especially in HIV-positive patients.

## Introduction

Tuberculosis (TB) is caused by *Mycobacterium tuberculosis* bacillus, which typically infects the lungs but can also affect virtually any organ of the body. TB is one of the top 10 causes of death worldwide and the leading infectious disease, above HIV/AIDS. Among patients with HIV, the estimated incidence in 2019 was 8.2% [1]. Among extrapulmonary TB, involvement of the liver is rare, presenting with nonspecific clinical and image manifestations that can lead to diagnostic delay [2]. The most common form of tuberculous involvement of the liver is as part of miliary (disseminated) TB disease, but it can manifest as localized (granulomatous) disease as well [3].

We present a case of hepatic tuberculosis in an HIV-positive male as part of a miliary form of the disease, with no signs of disseminated or pulmonary disease at first, reinforcing the importance of clinical suspicion of this rare form of TB.

## Case presentation

A 31-year-old male with previously known HIV and TB infection, nonadherent to antiretroviral (ARV) and anti-TB treatments, presented to the emergency department with fever, weight loss, malaise, and muscle aches over the past two weeks. Clinical examination revealed a fully oriented, cachectic patient; blood pressure of 100/60 mmHg, heart rate of 101 bpm, respiratory rate of 30 rpm; icteric sclerae, widespread hyperpigmented macules on the skin, and jaundice. The abdominal examination showed hepatomegaly five centimeters under the inferior costal border and no signs of ascites. General muscle strength was 3/5 in his four extremities.

**Figure 1:**
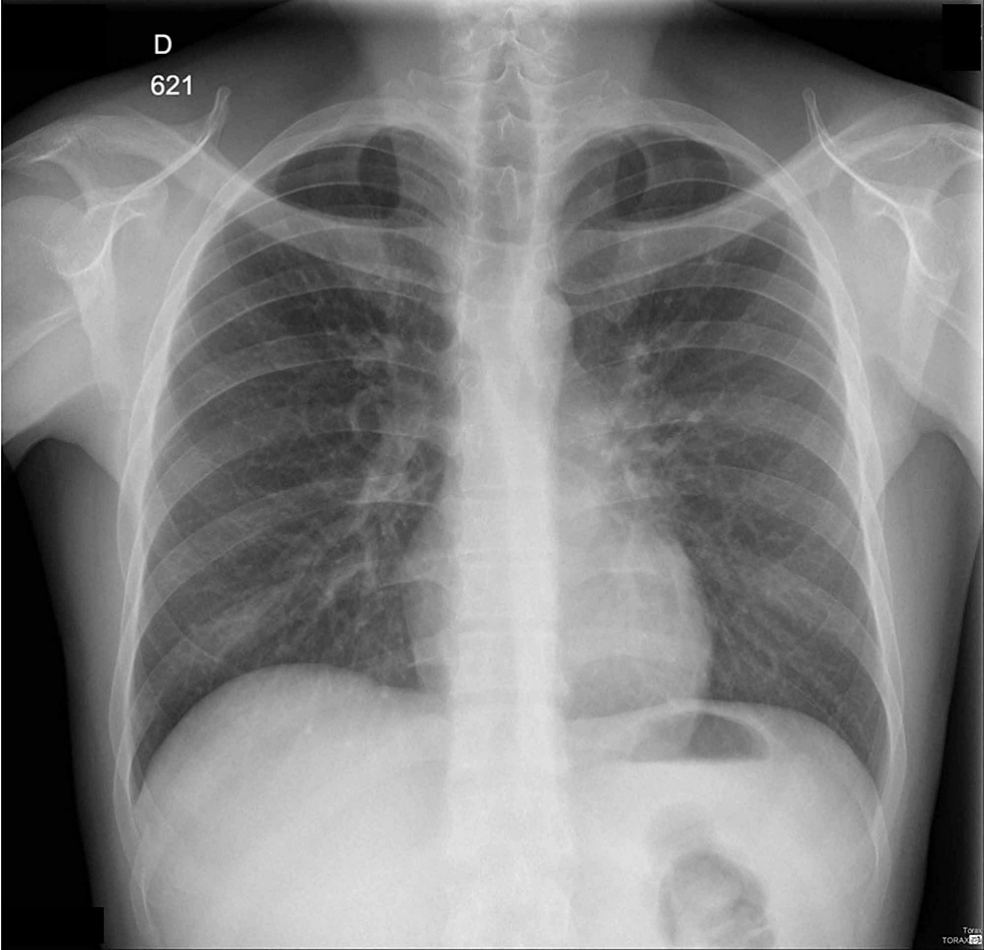
Chest X-ray on admission Chest x-ray on admission showing no infiltrates suggestive of pulmonary disease.

Further clinical evaluation revealed a CD4 (cluster of differentiation 4) lymphocyte count of 14 cells/µL, normocytic normochromic anemia with 8.9 g/dL of hemoglobin, and a normal platelet count of 185 000/µL. Liver function tests resulted as follows: aspartate transaminase (AST) = 1200 U/L, alanine transaminase (ALT) = 275 U/L, total bilirubin = 5.0 mg/dL, lactate dehydrogenase (LDH) = 1088 U/L, alkaline phosphatase (ALP) = 387 U/L, activated partial thromboplastin time (aPTT) = 48s, International Normalized Ratio (INR) = 2.0, the venereal disease research laboratory (VDRL) test = 1:512 dilutions (reactive), and antibodies against *Treponema pallidum *= 255. Serology tests for herpes simplex virus 2 (HSV), hepatitis C virus (HCV), and hepatitis B virus (HBV) were negative. Sputum analysis was negative for acid-fast bacilli. Chest x-ray on admission had no significant findings (Figure 1).

**Figure 2:**
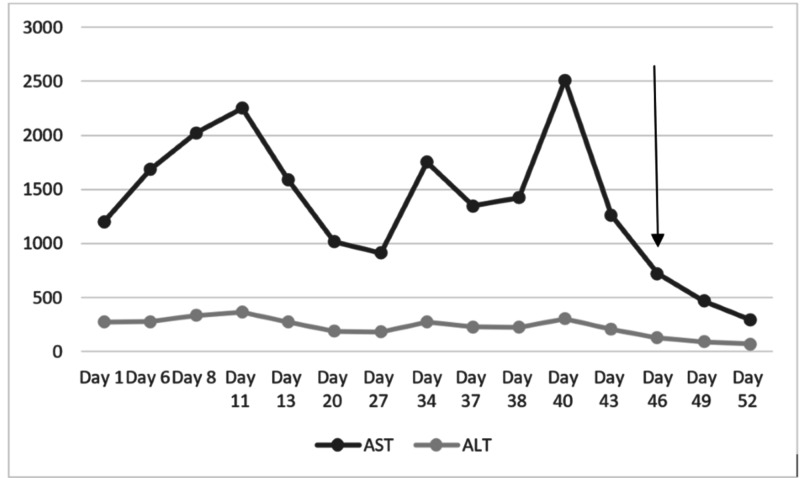
Evolution of liver enzymes throughout hospitalization Herein, we present the evolution of liver enzymes throughout hospitalization. The arrow represents the date of the biopsy. AST, Aspartate transaminase; ALT, alanine transaminase.

**Figure 3:**
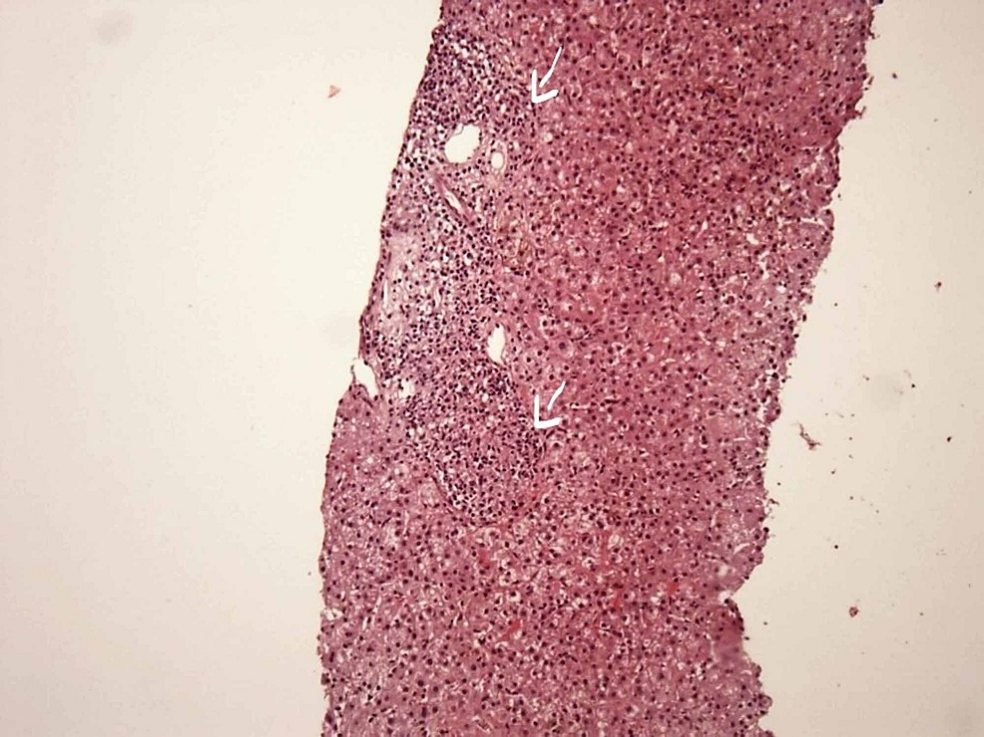
Light microscopy (10x) of the liver specimen with H&E staining Image showing the hematoxylin and eosin (H&E) staining of the liver specimen. Arrows show poorly defined microgranulomas.

**Figure 4:**
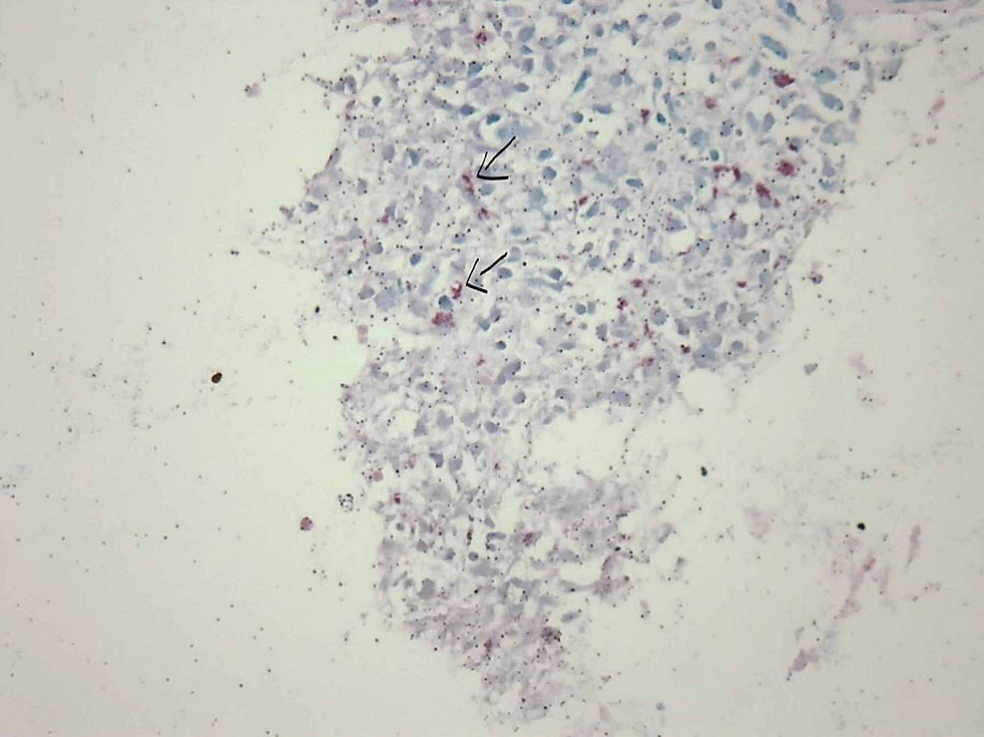
Light microscopy (10x) of the liver specimen with Ziehl-Neelsen staining Image showing Ziehl-Neelsen staining of the liver specimen. Arrows show acid-fast *Mycobacterium tuberculosis* bacilli.

**Figure 5:**
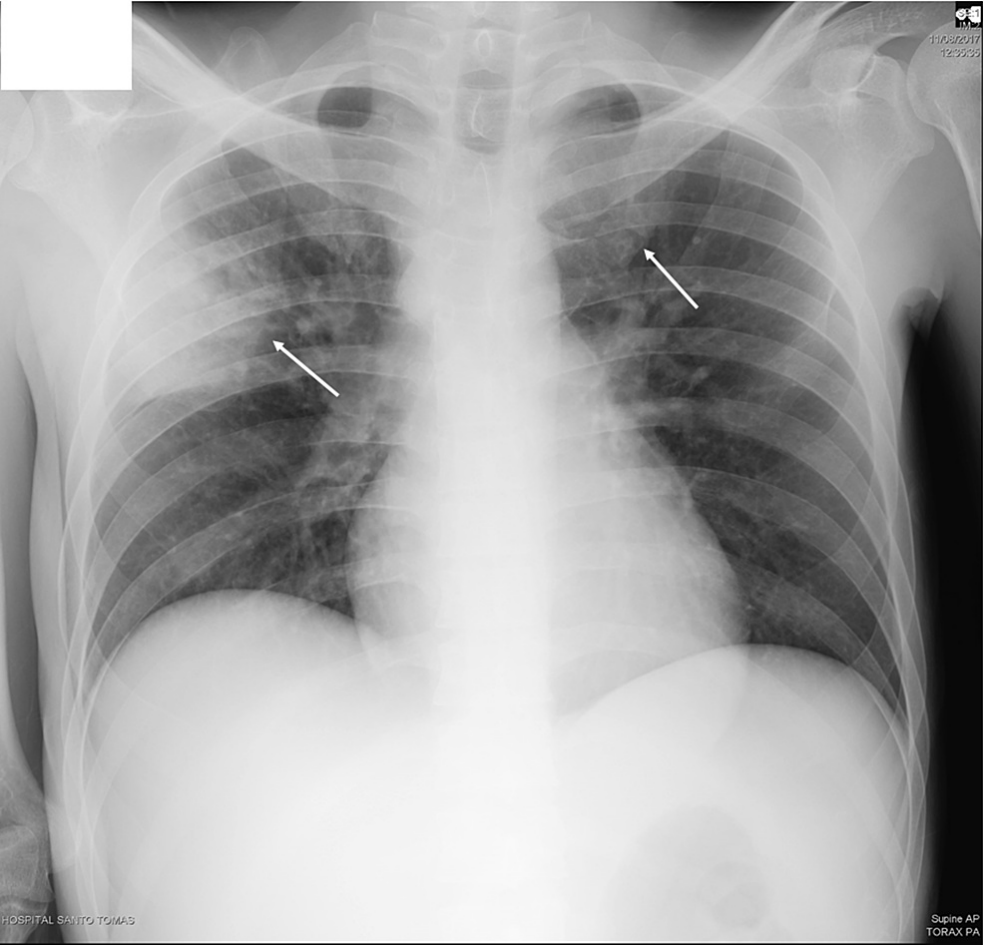
Chest x-ray on day 46 of hospitalization Image showing the chest x-ray after positive liver biopsy for *Mycobacterium tuberculosis*. The arrows show left lung alveolar infiltrates in the midpart of the lung and apical alveolar infiltrates in the right lobe.

After admission, an abdominal ultrasound was obtained, which revealed hepatomegaly; increased right liver lobe echogenicity; and enlarged, retroperitoneal, lymph nodes. Subsequently, benzathine penicillin G of 2.4 million units was administered for syphilis. By day 15 of hospitalization, the patient had partial relief of symptoms and a fall of the values of transaminases with supportive treatment alone. On day 30, his condition worsened with recurrence of abdominal pain accompanied by vomiting; liver enzymes started to increase, reaching 2000 U/L each and above (Figure 2). A computed tomography (CT)-guided liver biopsy was performed. The histopathologic analysis reported poorly defined granulomas (Figure 3). Ziehl-Neelsen stain of the liver sample showed acid-fast bacilli (Figure 4). A new chest x-ray showed alveolar infiltrates in the upper left and right lobes (Figure 5). The sputum analysis was again negative for acid-fast bacilli. GeneXpert test was run, resulting in positive for *Mycobacterium tuberculosis* and Rifampicin resistance. Standard bronchoscopy was not done due to the patient's clinical condition. 

The patient was diagnosed with disseminated tuberculosis involving the lung and liver. The patient was treated with the longer regimen as stated in National Tuberculosis Guidelines: intensive phase with pyrazinamide, isoniazid, levofloxacin, kanamycin, cycloserine, and ethionamide for six months, followed by 18 months of isoniazid, levofloxacin, cycloserine, and ethionamide. The patient had a good clinical headway, with an improvement of liver tests and resolution of jaundice and abdominal symptoms. Antiretroviral therapy (ART) with efavirenz/emtricitabine/tenofovir disoproxil fumarate was started two weeks after starting anti-TB therapy.

## Discussion

Hepatic TB is underreported in published literature. Prevalence and incidence are unknown. This is due to the unclear clinical presentation, rapid progression, and invasive nature of the precise diagnostic test. Conservative estimates of hepatic TB numbers have been made, resulting in an incidence of 1% of all TB cases and the prevalence of 50%-80% of liver involvement in patients with pulmonary TB [4]. Among the HIV/AIDS-infected individuals, the reports are limited to autopsies. Hepatic TB from the liver specimen of HIV-infected patients was seen in 17.4%, 23%, and 37.3% of autopsy reports in Taiwan, South Africa, and Nigeria, respectively. There is no consistent data on the mortality rate of hepatic TB in HIV-infected patients. In a case-control study of 20 HIV patients with hepatic TB, the mortality was 40% despite adequate treatment [4,5].

Hepatic TB is classified by one of the two methods: Reed or Levine. The former is a radiological classification that consists of three forms: hepatic TB associated with miliary TB, primary diffuse hepatic TB, and primary tuberculoma or abscess of the liver. The latter classification divides the disease into miliary TB, pulmonary TB with hepatic involvement, primary liver TB, focal granuloma or abscess, or tuberculous cholangitis [4]. In our case, it is more acceptable to adopt Levine’s classification since it specifies the pulmonary TB status of the patient, which was of vital importance since the resistance of the Mycobacterium was assessed with sputum analysis.

Presentation of hepatic TB is variable with nonspecific clinical and biochemical findings, which can hinder and delay diagnosis. Among case reports, fever, respiratory symptoms, abdominal pain in the right upper quadrant, loss of weight, malaise, night sweats, and jaundice are described in the clinical history. Hepatomegaly and ascites are the most described signs in the physical examination [2-6]. The most frequent laboratory findings are moderate or marked elevated bilirubin, elevated alkaline phosphatase, low serum albumin levels, normal or prolonged prothrombin time, and moderately elevated or normal aminotransferases [6]. These findings are not specific to hepatic TB since they can be present in any other liver disease. In the case presented, the increased aminotransferases are of interest that peaked up to 2500 U/L. The rest of the clinical history and findings in the physical exam were congruent with the reviewed literature.

Image studies such as x-rays, abdominal ultrasound, and abdominal CT can help in the diagnosis of hepatic TB. Plain x-rays and ultrasound of the abdomen are the most widely available and cost-effective image studies, but they lack specificity. In abdominal x-rays, calcified nodules may be present [3-6]. A TB suggesting chest x-ray may redirect the differential diagnosis toward hepatic TB. Approximately 75% of patients with hepatic TB have a TB suggesting chest x-ray [3]. The ultrasound often shows hypoechoic lesions with a bright liver pattern if the nodules are between 0.5 and 2 mm and complex masses with calcification if the lesion is bulkier. The most informative imaging test for hepatic TB is a contrast-enhanced CT scan of the abdomen, which shows low attenuation lesions with central enhancement in miliary TB and hypodense or calcified lesions with or without peripheral rim with central enhancement for hepatic tuberculoma [4-7]. MRI shows hypointense nodules with the hypointense rim on T1 and isointense or hyperintense with less intense or no rim on T2 [4]. MRI is less cost-effective than CT; therefore, the latter study is preferred in TB endemic areas [4]. In the case presented, an abdominal ultrasound revealed only retroperitoneal lymphadenopathies with an enlarged, hyperdense liver. Although contrast-enhanced CT was not done in our patient, the biopsy was CT-guided, and no masses were seen in the simple phase. Given the nonspecific findings in the imaging studies, the only diagnosis that could be made was hepatitis of unknown etiology, leaving an open compass of diseases that can cause hepatocellular damage. This complicated the picture even more since our patient was HIV-positive.

The diagnosis of hepatic TB is made by demonstrating caseating granulomata, confirmed by epithelioid cells, lymphocytes, and Langhans giant cells, or by demonstrating the presence of acid-fast bacilli of biopsy specimen [4-8]. CT-guided fine-needle aspiration biopsy is preferred over percutaneous puncture biopsy guided by ultrasound since the former offers better visualization of the liver anatomy, high puncture success rate, and lower complications such as bleeding and infection [8]. In the case reported, there were poorly differentiated microgranulomas. Specific stains for M. tuberculosis were done, given the background of the HIV status of the patient with a low CD4 count.

The differential diagnosis for hepatic TB in the patient among HIV-infected patients can be summarized into three major groups: opportunistic infections, concomitant infections due to virus or bacteria, and neoplasms that are related to medication [9]. Mycobacteria are one of the most common opportunistic infections. Fungal pathogens such as *Cryptococcus neoformans*, *Histoplasma capsulatum*, *Bartonella henselae*, and HSV hepatitis have been reported; nevertheless, serology for these causal agents was dismissed in our patient. Among the second group, the most common associated infections are HBV, HCV, and cytomegalovirus (CMV). Syphilitic hepatitis has been described, but there are only a few case reports. Hepatitis due to syphilis could have been an alternative diagnosis to the case presented, given the high VDRL titles (1:512) and the positive treponemal test. It presents with nonspecific symptoms such as right upper quadrant pain, hepatomegaly, and elevation of liver enzymes with an extremely elevated alkaline phosphatase. However, the patient had deleterious progress despite receiving adequate treatment. The latter two causes of hepatitis can be easily discarded in the case presented since there were no masses and the patient was nonadherent to antiretroviral (ARV) treatment.

Management of hepatic TB is based on standard anti-TB therapy regimens. Good clinical outcomes have been reported in liver abscess drainage in cases of localized tuberculomas [3]. The WHO treatment guidelines for drug-resistant TB, last updated in 2019, regroups the medicines into four groups: A to C and other medicines that are not included in groups A-C. The duration of therapy is also disserted in the WHO treatment guidelines for drug-resistant TB in 2019. They propose a shorter and longer regimen for adults and children. The shorter regimen consists of nine to 12 months and is recommended for people not previously treated with second-line drugs and in whom resistance to fluoroquinolones and second-line injectable agents is excluded or considered unlikely. However, these recommendations were done based on studies that only included pulmonary TB ill patients, and they cannot be extrapolated to extrapulmonary drug-resistant TB. Thus, for the case presented, the longer treatment recommendation is to be followed. The recommendation of the longer treatment is to start with pyrazinamide and four core second-line TB medicines, one from Group A, one from Group B, and at least one from Group C; they also recommend strengthening the regimen with high-dose isoniazid and/or ethambutol. The treatment does not change for people living with HIV. They do not make special considerations with hepatic TB. The composition is variable depending on the drugs available per country [10].

The patient from the case presented was treated with the longer regimen as follows: intensive phase with pyrazinamide, isoniazid, levofloxacin, kanamycin, cycloserine, and ethionamide for six months, followed by 18 months of isoniazid, levofloxacin, cycloserine, and ethionamide.

Drug regimens for resistant TB assure greater toxicity than first-line regimens, which prompts close monitoring for side effects. Liver function, thyroid function, serum chemistries, and audiometry screenings should be made. Hepatic TB along with the known hepatotoxicity of TB treatment can pose a challenge for patients with this particular situation. Nevertheless, it has been proven that hepatotoxicity of drug-resistant TB, specifically multidrug-resistant TB (MDR-TB) is seen in 16.5% of patients. The risk increases in patients with increased AST and ALT values at baseline. In retrospective case series, the meantime to hepatotoxic events was found to be from 196 to 231 days; however, these studies were done predominantly on pulmonary TB case series [11]. There is no single standardized recommendation on the time for liver function screening timing.

Another special consideration when treating patients with [hepatic] TB is the HIV status. There is a potential for TB-associated immune reconstitution inflammatory syndrome (IRIS) if antiretroviral therapy (ART) is initiated too early [1]. ART is recommended to be started within two weeks after TB treatment initiation when CD4 cell count is less than 50 cells/mm^3^ in patients who were not previously receiving ART treatment [12]. In the case presented, this recommendation was punctually followed as the patient was started on efavirenz/emtricitabine/tenofovir disoproxil fumarate two weeks after initiation of the mentioned anti-MDR-TB drug regimen. The patient did not present signs or symptoms of TB-associated IRIS or increased hepatotoxicity. Contrarily, the patient showed a decrease in AST, ALT, alkaline phosphatase, and LDH values, as shown above.

## Conclusions

Hepatic TB should be considered as a differential in an HIV patient presenting with hepatitis, even when the primary pulmonary disease is not evident. This is especially relevant in the picture of a low CD4 cell count, previous pulmonary TB, and nonadherence to ARV and TB treatment. Given the impaired T cell-mediated immunity of HIV/AIDS patients, clinical manifestations in the initial evaluation of disseminated TB disease can be nonspecific. Image studies such as ultrasound and abdominal contrast-enhanced CT can be useful, but they lack specificity and may give little information on the exact etiology of the hepatic disease. The diagnosis of hepatic TB is made by histopathologic demonstration of caseating granulomas or acid-fast bacilli. The treatment of hepatic drug-resistant TB in HIV/AIDS patients is the same as the one used in patients without HIV/AIDS. Hepatotoxicity and TB-IRIS are the most common complications of therapy but can be avoided with monitoring of liver function tests and initiation of ARV therapy at least two weeks after anti-TB therapy.
